# Pitfalls in Challenging Thyroid Tumors: Emphasis on Differential Diagnosis and Ancillary Biomarkers

**DOI:** 10.1007/s12022-020-09638-x

**Published:** 2020-07-06

**Authors:** José Manuel Cameselle-Teijeiro, Catarina Eloy, Manuel Sobrinho-Simões

**Affiliations:** 1 Department of Pathology, Clinical University Hospital, Health Research Institute of Santiago de Compostela (IDIS), Galician Healthcare Service (SERGAS), Santiago de Compostela, Spain; 2 Medical Faculty, University of Santiago de Compostela, Santiago de Compostela, Spain; 3 Department of Anatomic Pathology, Clinical University Hospital, Travesía Choupana s/n, 15706 Santiago de Compostela, Spain; 4 i3S Instituto de Investigação e Inovação em Saúde, Porto, Portugal; 5 Institute of Molecular Pathology and Immunology, University of Porto, Porto, Portugal; 6 Medical Faculty, University of Porto, Porto, Portugal; 7 Department of Pathology, Centro Hospitalar S. João, Porto, Portugal

**Keywords:** Thyroid, Rare tumors, PTC variants, FTC variants, Differential diagnosis, Molecular pathology

## Abstract

Thyroid pathology encompasses a heterogenous group of clinicopathological entities including rare and diagnostically challenging neoplasms. The review is focused on morphological, immunohistochemical, and molecular features of rare thyroid neoplasms that can pose diagnostic problems. The tumors are organized based on growth patterns including thyroid neoplasms with predominantly papillary, follicular, solid, and spindle cell growth pattern, as well as neoplasms with distinct cytological characteristics. A special section is also dedicated to rare thyroid tumors with peculiar patterns including thyroid carcinoma with Ewing family tumor elements and intrathyroidal thymic-related neoplasms.

## Introduction

Thyroid pathology encompasses a heterogenous group of entities including rare and diagnostically challenging neoplasms [1, 2]. In this review, the authors provided a diagnostic approach to rare thyroid tumors by taking into consideration morphological and immunohistochemical features as well as relevant molecular data. This helped the authors to review differential diagnostic problems. The clinicopathological diagnostic entities included rare cytomorphological variants of thyroid neoplasms as defined in the 2017 WHO classification [2] and also thyroid neoplasms that can pose challenges due to variations in cytomorphology (e.g., degree and amount of heterogeneity) and/or unusual immunohistochemical profiles in association with differentiated thyroid carcinoma (papillary thyroid carcinoma; PTC, or follicular thyroid carcinoma; FTC). This review did not focus primarily on details of non-invasive thyroid follicular neoplasms with papillary-like nuclear features as well as poorly differentiated and undifferentiated (anaplastic) thyroid carcinomas; however, relevant pitfalls are provided during the discussion of differential diagnoses.

## Tumors with Predominant Papillary Growth Pattern

### Columnar Cell Variant of Papillary Thyroid Carcinoma

**Fig. 1:**
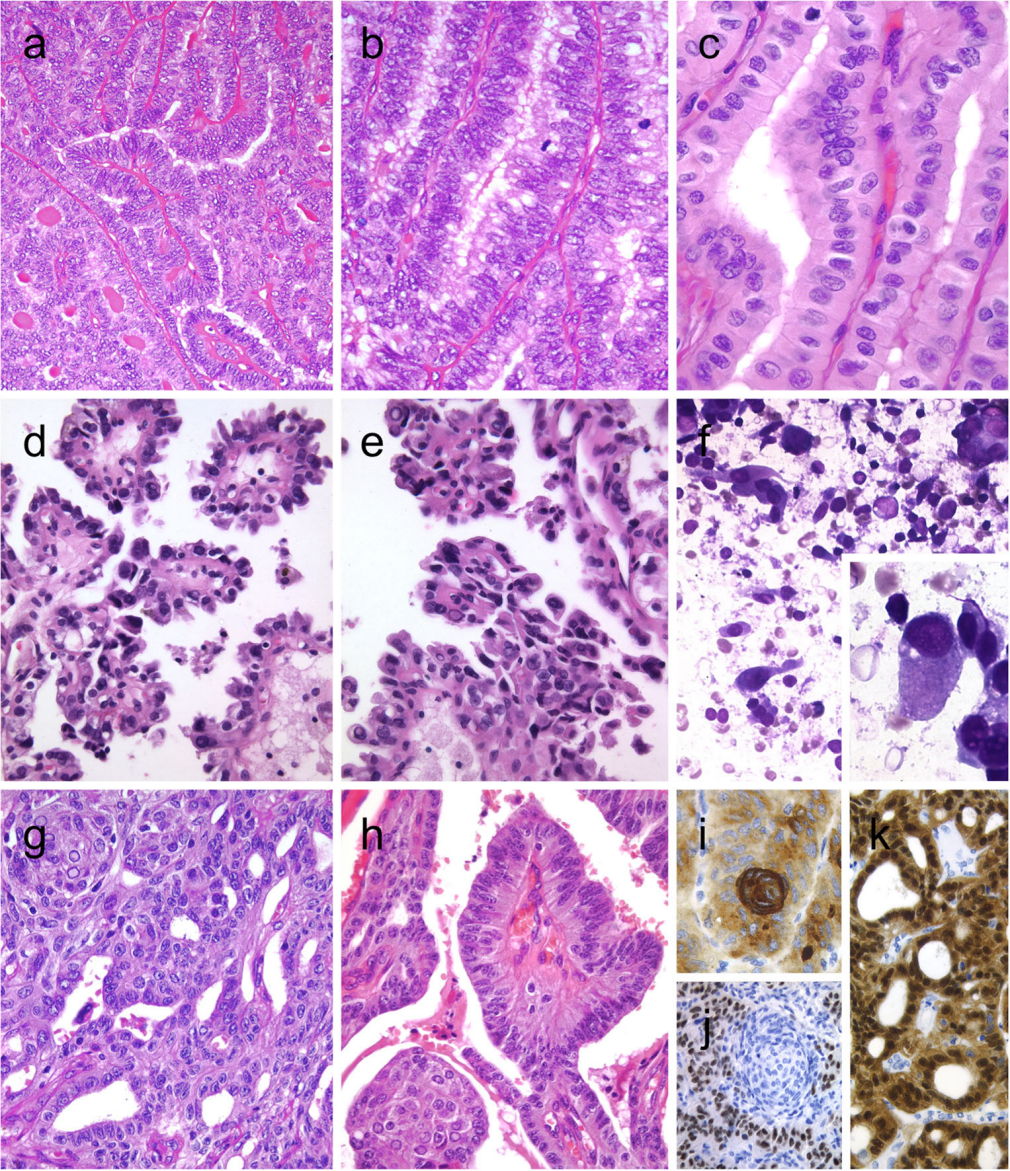
Columnar cell variant of papillary thyroid carcinoma (PTC) showing a combination of papillary and glandular-like patterns (**a**), marked nuclear pseudostratification, and less nuclear features of classic PTC (**b**). In the tall cell variant of PTC, the cytoplasm is deeply eosinophilic, and nuclear features of PTC are very prominent with irregular contours and common pseudoinclusions (**c**). Hobnail variant of PTC combining papillary (**d**), and micropapillary (**e**) structures lined by hobnail cells. “Teardrop” cells (**f**) and comet-like cells (inset). The cribriform-morular thyroid carcinoma exhibits a blending of cribriform, papillary, trabecular, and solid pattern with morules (**g**) and (**h**). Morules are strongly positive for CD10 (**i**)**.** Tumor cells are reactive for estrogen receptors (**j**), and there is strong nuclear and cytoplasmic reactivity for β-catenin (**k**)

Columnar cell variant of PTC consists predominantly of columnar cells that are characterized by marked nuclear pseudostratification [2–4]. This variant shows a combination of papillary, glandular-like, and/or solid growth patterns (Fig. 1a and b). Unlike tall cell variant of PTCs (Fig. 1c) that often exhibit an eosinophilic-glassy cytoplasm and basally located nuclei with well-developed (florid) nuclear features of PTC, columnar cell variants often display subnuclear vacuolization and/or cytoplasmic clearing along with elongated nuclei, dark chromatin, and less florid nuclear features of classic PTCs. For this reason, it is generally considered that columnar cell variants of PTCs are reminiscent of an endometrioid or intestinal adenocarcinoma of various sites. Occasionally, this diagnosis can be rendered on fine needle aspiration biopsy (FNAB) specimens [5]. While there are also PTCs consisting of mixed columnar cell and tall cell variants, combined tumors in association with mucoepidermoid carcinoma and columnar cell variants with hyaline globules that can simulate adenoid cystic carcinoma have been recognized [6]. Columnar cell variants of PTCs have also significant morphologic overlap with the cribriform-morular thyroid carcinoma (also known as cribriform-morular variant PTC in the 2017 WHO classification).

By immunohistochemistry, columnar cell variants are typically immunoreactive for TTF1, PAX8, thyroglobulin, estrogen and progesterone receptors, and cyclin D1. Membranous β-catenin staining distinguishes these tumors from cribriform-morular thyroid carcinomas [7]. There is often weak nuclear p53 staining along with elevated Ki-67 proliferation index. Calcitonin and carcinoembryonic antigen (CEA) are negative [4, 6, 8]. Positivity for CDX2, a putative intestinal-type transcription factor, has been reported in some cases [7–9]. The latter can confuse the diagnostician, but the positivity for TTF1, PAX8, and thyroglobulin solves this quandary. Immunoreactivity for β-human chorionic gonadotropin was also reported in a case [10]. Unlike tall cell and classical variants, *BRAF*^V600E^ mutation is relatively less common and has been reported in about one third of cases [8, 11].

Traditionally, these neoplasms have been considered as an aggressive form of PTC; however, evidence also suggested that infiltrative forms are associated with adverse outcome when compared with their encapsulated counterparts [12].

### Hobnail (Micropapillary) Variant of Papillary Thyroid Carcinoma

Hobnail variant of PTCs are defined when at least 30% of the tumor volume shows hobnail cell change [2, 13, 14]. This variant is also designated as a micropapillary variant of PTC [2], because these tumors exhibit sometimes a combination of papillary and/or follicular growth pattern with a predominance of micropapillary structures covered by hobnail cells (Fig. 1d and e). It is important to distinguish hobnail cell variants from a cystic degenerate classical variant PTCs with hobnail cell-like change. Hobnail cells are cuboidal or elongated follicular cells with eosinophilic cytoplasm. Apically placed large nuclei with distinct nucleoli give their characteristic appearance. Tumor cells have an increased nuclear to cytoplasmic ratio, pleomorphic nuclei, and moderate to severe nuclear atypia. Diagnostic nuclear features of classic PTC are usually seen. This diagnosis can also be suspected on FNAB specimens [15] (Fig. 1f). Psammoma bodies and variable mitoses, including atypical forms can be seen. The mean Ki-67 labeling index has been reported as 10% [13, 14]. Necrosis, lymphatic and vascular invasion, and extrathyroidal extension are not rare [16, 17]. Concomitant tall cell areas were seen in around 40% of cases [16]. Hobnail cell variants with columnar cell change were also reported [18, 19]. Synchronous or metachronous association of this variant with poorly differentiated thyroid carcinoma [19, 20] or with anaplastic thyroid carcinoma [18, 19] has also been described.

The overall immunohistochemical profile of this cytological variant of PTC is not much different than that of the classic PTC [18]. However, these tumors show strong nuclear staining for p53 in most cases [13, 16, 18, 19, 21, 22].

*BRAF*^V600E^ and *TP53* mutations are the most common molecular alterations in this variant of PTC [18, 19, 21, 23, 24]. *TERT* promoter mutations [18, 23, 24], *PIK3CA*, *CTNNB1*, *EGFR*, *AKT1* and *NOTCH1* mutations [24], and *RET/PTC1* rearrangements [19] have also been reported.

Hobnail cell variant of PTCs are well recognized for their biological aggressivity. In addition, PTCs with 10% hobnail and/or micropapillary features have also been linked to a poor outcome [25]. Therefore, similar to tall cell variant of PTCs, focal hobnail cell change (less than 30%) should also be documented in the pathology report. The latter is of significance as a potential pitfall would be not to call classic PTCs with ischemic/degenerative hobnail cell-like changes as PTCs with focal hobnail cell change, as such tumors lack aggressive histopathological features and pursue an indolent clinical course [26].

### Cribriform-Morular Thyroid Carcinoma

In the 2017 WHO classification, this tumor was classified as a variant of PTC as cribriform-morular variant [2]; however, there is a growing evidence suggesting that these tumors do not belong to the PTC family [27]. These tumors can be associated with familial adenomatous polyposis (FAP), but sporadic manifestations also occur [2]. In FAP patients, these tumors are usually multifocal and bilateral, whereas in sporadic manifestations, solitary neoplasms predominate [27, 28]. These tumors are often encapsulated or well delineated with variable mixture of complex architecture including cribriform, papillary, follicular, trabecular and solid patterns, as well as morular structures (Fig. 1g and h). The morules lack keratinization and consist of some cells with peculiar (biotin-rich) nuclear clearing and can be selectively stained for CDX2 and CD10 (Fig.1i). Tumor capsular invasion and angioinvasion have been reported in about 40% and 30% of cases, respectively.

By immunohistochemistry, the tumor cells are often negative but can be focally positive for thyroglobulin; however, they are positive for TTF1, PAX8 (variable staining intensity), and estrogen (Fig. 1j) and progesterone receptors and are negative for CK20 and calcitonin. A strong nuclear and cytoplasmic reactivity for β-catenin (Fig. 1k) is the hallmark of this tumor [1, 7, 27, 28]. LEF-1 has also been suggested as a sensitive biomarker for cribriform-morular thyroid carcinomas in a recent series [29]; however, the global experience is largely lacking with respect to LEF-1 expression in these neoplasms. Odd cases with positivity for chromogranin and synaptophysin [30], as well as for β-hCG, have also been reported [31]. FNA samples can be diagnostic in some cases [1, 27].

The peculiar endodermal (intestinal-like) tumor phenotype is due to the permanent activation of the WNT/β-catenin pathway secondary to germline and/or somatic mutations in *APC*, *CTNNB1*, and/or *AXIN1* [27, 32]. *RET/PTC* rearrangements and mutations in *PIK3CA* or *RAS* genes can act as additional upstream effectors in this pathway in sporadic and FAP-associated cribriform-morular thyroid carcinoma [27]. Because of this distinctive genotype-phenotype correlation and clinicopathological findings, this tumor has been proposed as a type of thyroid tumor in itself rather than a subtype of PTC [27]. Due to its cytoarchitectural pattern, frequent thyroglobulin negativity, and estrogen and progesterone receptor positivity, these tumors can be mistaken for metastatic carcinoma of breast or colorectal origin. However, positivity for TTF1 often facilitates the appropriate diagnosis. There is morphological overlap between the cribriform-morular thyroid carcinoma and columnar cell variant of PTCs. In addition to previously discussed cytomorphological pitfalls (see columnar cell variant of PTC), absence of morules, frequent positivity for thyroglobulin, and absence of nuclear beta-catenin expression distinguish these tumors from cribriform-morular thyroid carcinomas. Although the solid growth pattern in cribriform-morular thyroid carcinoma can simulate poorly differentiated carcinoma, a characteristic cribriform pattern with morules and lower mitotic index can help in this distinction. Occasionally, lung metastases of cribriform-morular thyroid carcinomas can simulate primary pulmonary adenocarcinoma, particularly if the immunohistochemical panel is limited [33].

Cribriform-morular thyroid carcinomas are generally thought to portend a favorable prognosis [1, 27], but those with neuroendocrine differentiation [30], tumors with dedifferentiation to poorly differentiated thyroid carcinoma, and/or *TERT* promoter mutations [34] have been associated with an aggressive clinical course. In addition, those with a high Ki-67 index but unassociated with mitotic activity do not seem to represent a poor prognostic category [35]. Another important implication of this diagnosis is related to the need for further screening for the possibility of FAP-related germline disease. Therefore, clinicians should be alerted to the possibility of FAP when a diagnosis of cribriform-morular thyroid carcinoma is made.

### Papillary or Pseudopapillary Variant of Medullary Thyroid Carcinoma

**Fig. 2:**
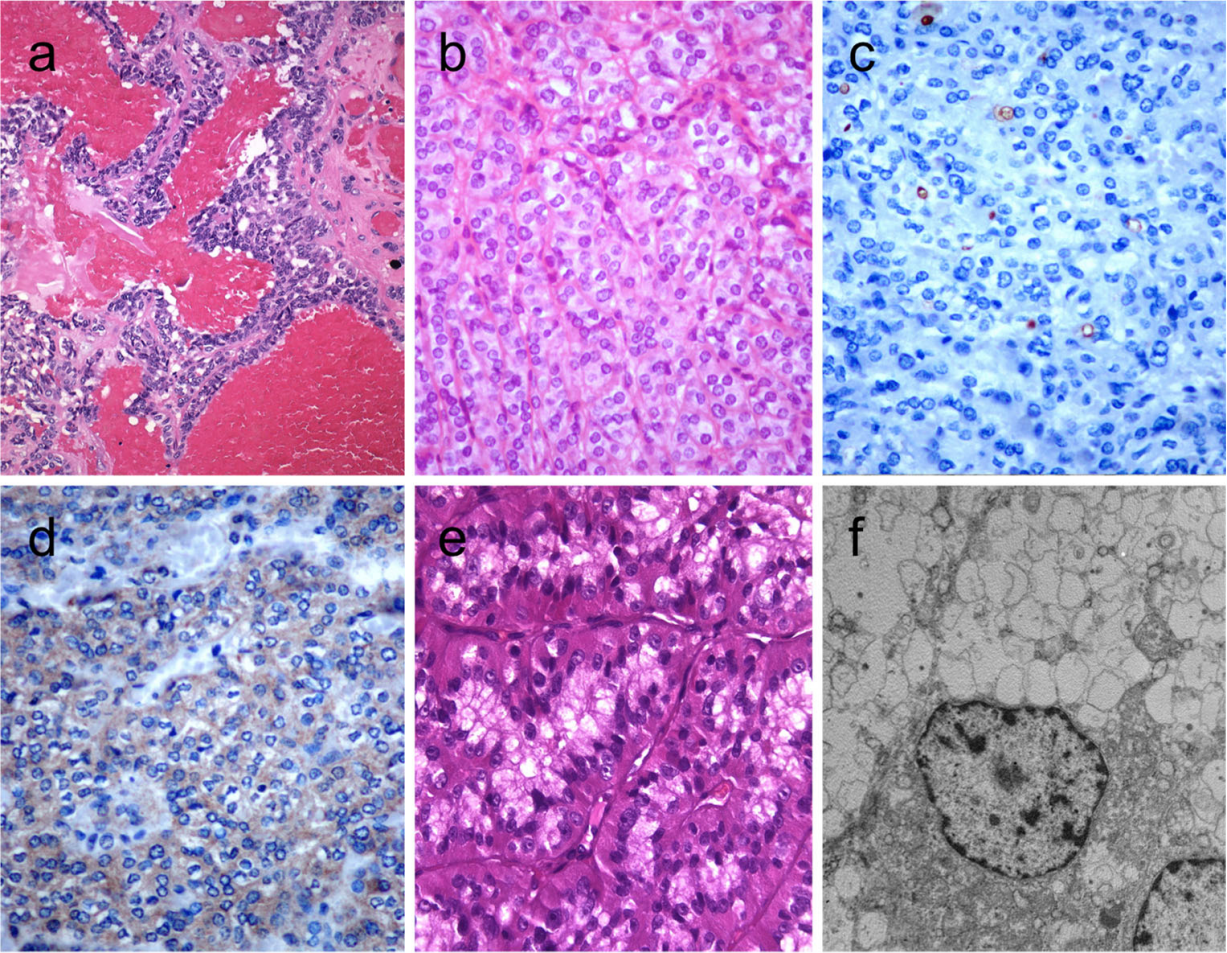
Medullary thyroid carcinoma with papillary pattern (**a**). Solid variant of papillary thyroid carcinoma (**b**) with focal expression of thyroglobulin (**c**) and expression of T4 (**d**); in this case, TTF1 was diffusely expressed. Biphasic Hürthle cell (oncocytic) clear carcinoma in which the basal half of the cytoplasm is oncocytic, whereas the upper half is clear (**e**), due to the swelling of the mitochondria (ultrastructure) (**f**)

Medullary thyroid carcinomas can exhibit pseudopapillary or papillary pattern either focally or exceptionally, throughout the nodule (Fig. 2a) [36]. In comparison with other variants of medullary thyroid carcinoma, those with papillary growth pattern seem to carry a good prognosis [37]; however, this has not been universally validated.

By immunohistochemistry, the tumor cells express calcitonin, calcitonin gene-related peptide (CGRP), and monoclonal CEA [7]. This immunoprofile distinguishes these tumors from follicular cell-derived thyroid carcinomas with papillary growth [36]. Medullary thyroid carcinomas may show a papillary pattern with a totally cystic gross appearance [36, 38].

While TTF-1 is typically expressed in most medullary thyroid carcinomas, one should also note that PAX8 antibodies can show reactivity depending on their clones; for instance, most C-terminus specific PAX8 antibodies and N-terminus-specific MRQ-50 PAX8 antibody are often negative in medullary thyroid carcinomas [39]. Failure to recognize this pitfall may result in misclassification of a papillary variant of medullary thyroid carcinoma as a follicular epithelial derived neoplasm [39].

### Villous Variant of Papillary Thyroid Carcinoma

This is an extremely rare cytomorphological variant of PTC that was reported in a patient with Marfan syndrome due to *FBN1* gene mutation [40]. The term villous is proposed by Winer et al. because of the tumor growth characterized by prominent long villous fronds that were unassociated with tall cell or columnar cell features [40].

The tumor can simulate a metastatic carcinoma; however, the demonstration of thyroglobulin, TTF1, and PAX8 confirms the thyroid follicular origin. At a molecular level, the reported tumor harbored pathogenic *BRAFV600E* mutation. Winer et al. hypothesized that the presence of *BRAFV600E* mutation in the background of TGF-β-related epithelial-to-mesenchymal transition with active phospho-SMAD signaling may have resulted in this peculiar morphology [40]. Although the data is sparse, the distinction of villous variant of PTC is of clinical interest as it may be a harbinger of Marfan syndrome.

### Papillary Thyroid Carcinoma with Classic Architecture and Reduced Thyroglobulin Expression

PTCs often display variable cytomorphological features. In solid, spindle, clear cell, and diffuse sclerosing variants, expression for thyroglobulin can be variable (Fig. 2b–d). Reduced or absent staining can also feature cribriform-morular and rarely in columnar cell variant of PTCs. Nevertheless, absence of thyroglobulin expression in a papillary thyroid carcinoma requires further assessment; first concerning the influence of less adequate preanalytical conditions and, second, for the possibility of a genetic defect, either somatic or constitutional, that compromise the integrity of the thyroglobulin as a protein. Combined reactivity for monoclonal PAX8 and TTF1 antibodies often support the thyroid follicular epithelial origin in the appropriate cytomorphological and clinical context [7]. However, the demonstration of thyroperoxidase and T4 expression or application of in situ hybridization to detect thyroglobulin RNA is also helpful in the identification of follicular differentiation.

## Tumors with Predominant Follicular Growth Pattern

### Follicular Neoplasms with Clear Cell Change

Focal clear cell change is not rare in thyroid nodules; however, clear cell thyroid tumors, defined by more than 50% of clear cells, are uncommon [2]. Optically clear cytoplasm results from an accumulation of glycogen, lipid, mucin, and thyroglobulin but can also occur due to dilatation of mitochondria (oncocytic clear cell tumors) or to distended Golgi complexes [2, 41].

Gain-of-function mutation of *TSHR* might also contribute to the development of clear cell appearance in thyroid tumors [42]. Clear cell variants of follicular adenoma/carcinoma, oncocytic cell tumors, and papillary and medullary thyroid carcinoma have been recognized in the 2017 World Health Organization classification [2]. Peculiar biphasic oncocytic and clear cell change characterized by oncocytic change in the lower half of the cytoplasm and clear cell change (due to swelling of mitochondria) in the remaining half of tumor cells have been reported in thyroid neoplasms [1] (Fig. 2e and f).

One should be aware of clear cell thyroid nodules within conventional thyroid tumors (tumor-in-tumor) that may represent metastatic renal carcinoma [43, 44]. Immunomarkers for follicular cells (thyroglobulin, TTF1, and PAX8), C cells (calcitonin, CGRP, and monoclonal CEA), and parathyroid tissue (parathyroid hormone, GATA3, and GCM2) can assist the correct diagnosis. Although TTF1 is also detected in lung tumors and PAX8 in kidney tumors, the combined expression of these two markers is strongly indicative of a thyroid follicular epithelial lineage [2]. Metastatic renal carcinomas can be identified on thyroid FNAB specimens [43].

Traditionally, clear cell variants of thyroid carcinomas have not been linked to any biological aggressiveness. However, an aggressive clear cell variant of follicular thyroid carcinoma combining a putative gain of function of *TSHR* and a loss-of-function *TP53* has also been reported [42].

### Functional Follicular Neoplasms

**Fig. 3:**
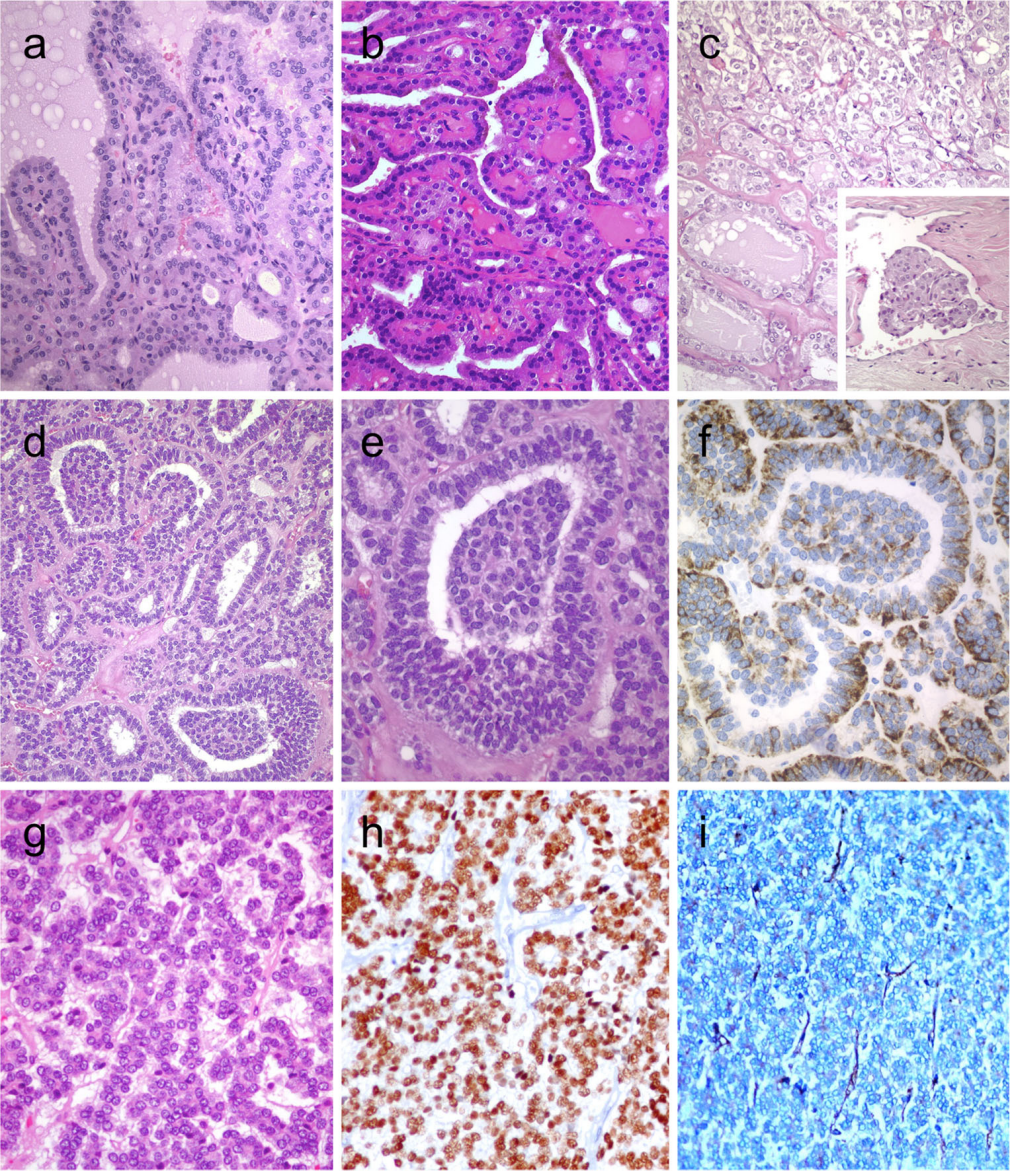
Hyperfunctioning follicular adenoma typically shows follicles with papillary infoldings and bubbly, pale colloid with peripheral scalloping (**a**). Non-hyperfunctioning adenomas with papillary hyperplasia usually show a more predominantly papillary pattern without vacuolated cytoplasm and scalloping colloid (**b**). Rare hyperfunctioning follicular tumors (**c**) can show capsular and/or venous invasion (inset); the nuclei are very clear which may be associated to hyperfunctioning. The glomeruloid pattern in this follicular thyroid carcinoma (FTC) included follicles with round to oval tufts growing within, at times supported by a fibrovascular core mimicking the renal glomerulus (**d** and **e)**; empty follicles were lined by columnar cells with marked pseudostratification, and positivity for CK18 was detected (**f**). FTC (**g**) with TTF1 expression (**h**) and very focal expression of thyroglobulin **i**

Functional follicular thyroid tumors are warm or hot on thyroid scans. Most of these nodules are follicular adenomas that exhibit follicular growth and variable intrafollicular centripetal pseudopapillary projections, papillary infoldings, and bubbly, pale colloid with peripheral scalloping (Fig. 3a). The cells are tall and display abundant eosinophilic to vacuolated cytoplasm. The uniform round nuclei are basally located. Nonfunctional follicular thyroid adenomas with papillary hyperplasia show a more predominantly papillary pattern of growth with cystic areas. The latter lacks vacuolated cytoplasm and scalloping of colloid (Fig. 3b).

The most important differential diagnosis of a functional follicular adenoma is the encapsulated tall cell variant of PTC in which elongated and tightly packed follicles and papillae are lined by tall cells with dense eosinophilic cytoplasm. The presence of florid nuclear features of PTC and loss polarity of nuclei are useful in this distinction (Fig. 1c).

At a molecular level, functional follicular adenomas have been linked to activating mutations in *TSHR*, *GNAS*, and/or *EZH1* genes [45, 46]. In most cases, *EZH1* gene mutation has been detected in association with either *TSHR* or *GNAS* mutations, suggesting a 2-hit model for the pathogenesis of autonomous thyroid adenomas [46]. Although the rate of malignancy is traditionally less frequent in functional thyroid nodules, autonomous nodules can also be malignant [47]. Most cases carry mutations in the TSHR signaling pathway; cases with concurrent mutations in *TSHR* and *RAS* genes [48], as well as concomitant *TSHR* mutation and *PAX8-PPARγ* rearrangement, have been reported [47] (Fig. 3c). Metastatic lesions may also preserve the autonomous production of thyroid hormone [49]. A recent series reported an increased risk of malignancy in *TSHR*-mutant functional thyroid nodules when *TSHR* mutations occur at high allelic frequency [50].

### Follicular Tumors with Unusual Growth and/or Immunohistochemical Profile

Follicular thyroid neoplasms can display unusual growth pattern. A rare follicular thyroid carcinoma with an unusual glomeruloid pattern of growth has been reported [51]. This neoplasm was widely infiltrative with tumor nodules infiltrating the adjacent thyroid tissue, displaying several foci of vascular invasion. The peculiar growth pattern included follicles with round to oval tufts growing within, at times supported by a fibrovascular core mimicking renal glomeruli (Fig. 3d–f). For this reason, the tumor has been referred to a glomeruloid variant of FTC. The tumor cells were immunoreactive for TTF1, thyroglobulin, thyroperoxidase, CK18 (Fig. 3f), HBME-1, and vimentin. Scattered cells were also positive for Wilms tumor 1 (WT1) and pankeratin (clone AE1/AE3); there was negativity for CK7 and CK19. Both *PAX8-PPARγ* rearrangement and *NRAS* mutations were detected. Glomeruloid variant of follicular thyroid carcinoma can be mistaken for cribriform-morular thyroid carcinoma, but lack of CDX2 and CD10-positive morules and absence of nuclear β-catenin positivity can help in this distinction. At variance with poorly differentiated thyroid carcinoma, glomeruloid variant of FTC was reported to have a low proliferative index and no necrosis [51]. When compared with a metastatic nephroblastoma (Wilms tumor), positivity for thyroglobulin and TTF-1 and only cytoplasmic and very limited positivity for WT1 distinguish this tumor from metastatic Wilms tumor. More recently, a glomeruloid variant of follicular adenoma of the thyroid has also been reported [52].

As described in PTCs, the frequency of aberrant immunohistochemical profiles in follicular tumors is unknown (Fig. 3g–i). Thyroglobulin expression can also be altered in clear cell follicular thyroid neoplasms [53]. As discussed earlier, the glomeruloid variant of follicular carcinoma harbors an aberrant phenotype expressing WT-1, such as the tumors of the kidney [51].

### Follicular or Pseudofollicular Growth in Medullary Thyroid Carcinoma

**Fig. 4:**
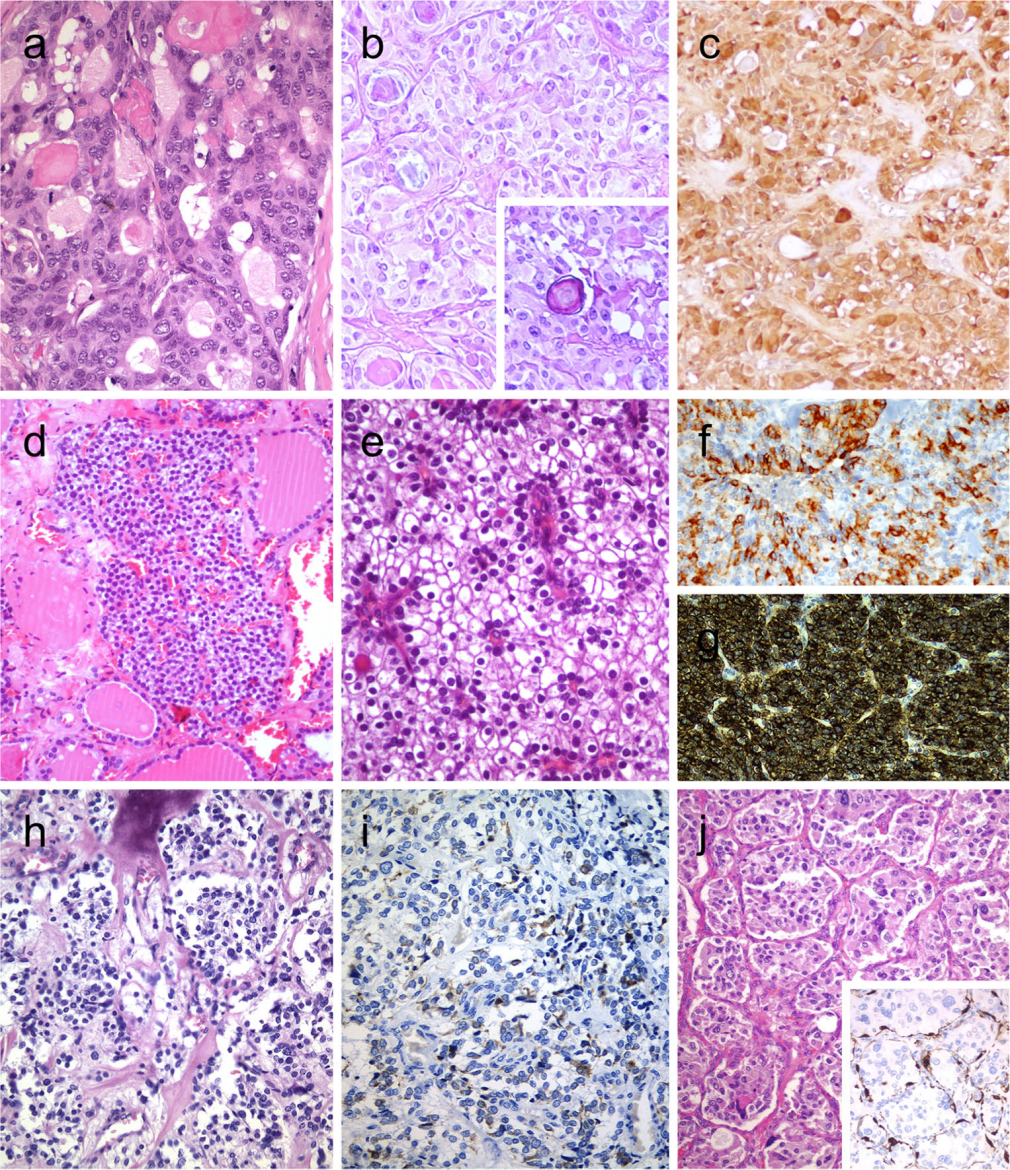
Follicular patterned medullary thyroid carcinoma (MTC) (**a**). In this other follicular patterned MTC (**b**), there are several calcifications simulating psammoma bodies (inset) and positivity for calcitonin (**c**). Intrathyroidal parathyroid tissue (**d**). The microscopic aspect of an intrathyroidal parathyroid adenoma is similar to eutopic parathyroid adenomas (**e**). Intrathyroidal parathyroid adenoma expressing chromogranin A (**f**) and PTH (**g**). Calcitonin-negative medullary thyroid carcinoma (**h**) showing positivity for CGRP (**i**). Paraganglioma (**j**) typically shows negativity for calcitonin and S100-positive sustentacular cells (inset)

Medullary thyroid carcinomas with true follicular growth are extremely rare. Similar to pseudopapillary growth, a pseudofollicular growth can occur more frequently due to lack of cohesiveness in medullary thyroid carcinomas. Medullary thyroid carcinomas with follicular growth can easily be passed unnoticed in the routine assessment due to the extreme resemblance to follicular nodular disease (Fig. 4a–c). These tumors also simulate a mixed medullary thyroid carcinoma and follicular cell-derived thyroid carcinoma. Positivity for calcitonin, CGRP, and monoclonal CEA in the tumor cells can assist the diagnosis [38, 54].

### Intrathyroidal Parathyroid Neoplasms with Follicular Growth

Parathyroid adenoma and carcinoma can manifest with an intrathyroidal nodule [2] (Fig. 4d). Intrathyroidal parathyroid adenomas are frequently misdiagnosed as follicular lesion on FNAB specimens. Intrathyroidal parathyroid adenoma (Fig. 4e) must not be interpreted as an evidence of parathyroid carcinoma. The diagnosis of intrathyroidal parathyroid carcinoma requires demonstration of invasive growth.

Positivity for chromogranin A, GATA3, GCM2, and PTH can distinguish parathyroid origin [2, 55, 56] (Fig. 4f–g). One should also be aware that parathyroid proliferations can display aberrant reactivity for calcitonin and CGRP; therefore, positivity for GATA3 and GCM2 and negativity for monoclonal CEA and TTF1 can be used to confirm the parathyroid origin [56].

## Tumors with Predominant Solid Growth Pattern

### Calcitonin-Negative Medullary Thyroid Carcinoma

The overwhelming majority of primary neuroendocrine tumors of the thyroid are medullary thyroid carcinomas that originate from C cells. Medullary thyroid carcinomas are almost always positive for monoclonal CEA but can be variably reactive for calcitonin, CGRP, and general neuroendocrine markers [2, 7].

The differential diagnosis of medullary thyroid carcinoma includes primary paraganglioma of the thyroid gland, intrathyroidal thymic neuroendocrine neoplasms, as well as metastatic neuroendocrine tumors to the thyroid [57]. Several neuroendocrine neoplasms including a subset of medullary thyroid carcinomas can show intratumoral sustentacular cells. Unlike most paraganglioma-like variant of medullary thyroid carcinoma (Fig. 4h and i), intrathyroidal paragangliomas tend to exhibit well-developed S100-positive sustentacular cells (Fig. 4j). While calcitonin and CGRP can also be expressed in paragangliomas, the demonstration of tyrosine hydroxylase and GATA3 distinguishes paraganglioma from other neuroendocrine neoplasms. TTF-1 and thyroglobulin are negative in paragangliomas.

One should be aware that thyroglobulin can cause diffusion-type staining in some cases; a few primary neuroendocrine thyroid tumors show concomitant positivity for neuroendocrine markers and thyroglobulin along with negativity for calcitonin and monoclonal CEA [58]. CGRP is generated as an alternative RNA splicing of the *CALCA* gene encoding calcitonin, CGRP, and catakalcin (procalcitonin). Our group and others [49, 50] have confirmed that positivity for CGRP also supports a C cell origin. A subset of unusual calcitonin-negative medullary thyroid carcinomas are characterized by no elevated serum calcitonin levels, no calcitonin expression on immunohistochemistry, and no expression for calcitonin mRNA. These tumors have been reported to express TTF1 and PAX8 (polyclonal), limited or no positivity for CEA, and negativity for thyroglobulin [59, 60]. *RET*, *H-RAS*, *KRAS*, or *BRAF* mutations were not detected in calcitonin-negative cases [59, 60]. Additional cases are needed to confirm the existence of primary high-grade neuroendocrine carcinomas (small or large cell types) of the thyroid with negativity for thyroglobulin, calcitonin, CGRP, and CEA [61, 62].

### Thyroid Tumor with Neoplastic Solid Cell Nest Features

Solid cell nests are remnants of ultimobranchial body remnants [63]. They are composed of main (chief) cells that are positive for p63, p40, high- and low-molecular-weight cytokeratins, galectin 3, Bcl-2, CEA, TTF1 (clone SPT24), and GATA3 and are negative for monoclonal PAX8 [63, 64]. C cells can sometimes be admixed with main cells of solid cell nests.

**Fig. 5:**
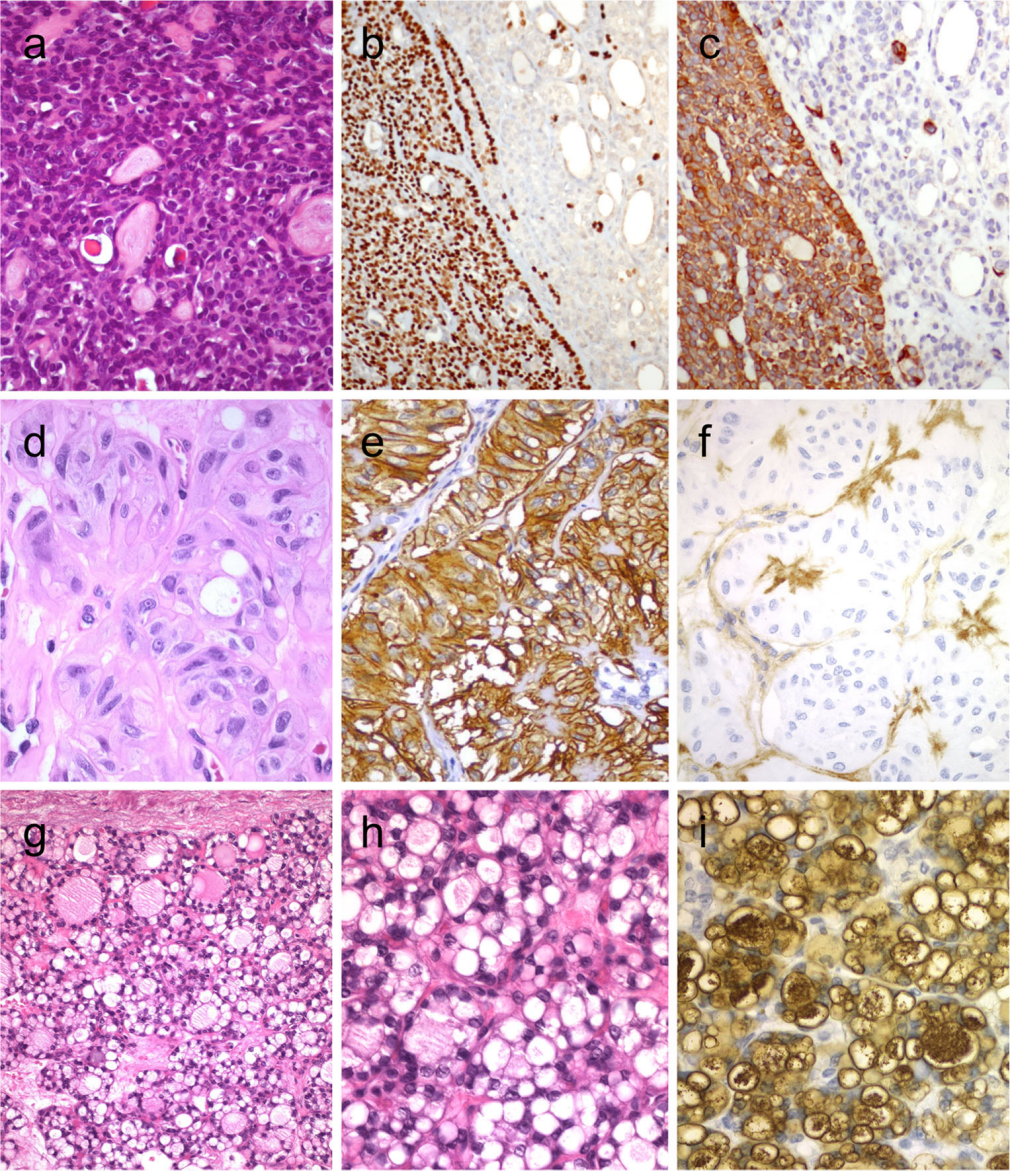
Tumor of the thyroid with solid cell nest features disclosing small cells of the main cell type (**a**) that express p63 (**b**) and cytokeratin 5 (**c**), in the absence of TTF1, calcitonin, and thyroglobulin expression. Hyalinizing trabecular tumor (**d**–**f**) is composed of trabeculae of elongated or polygonal cells admixed with abundant amounts of hyaline material negative for amyloid and positive for type IV collagen (**f**); Ki-67 is characteristically expressed in the cell membrane but not in the nuclei of the tumor cells (**e**). Follicular adenoma with signet ring cells (**g** and **h**), showing strong positivity for thyroglobulin (**i**)

Hyperplastic solid cell nests are not unusual in thyroids removed due to other causes, and giant solid cell nests have also been documented [65, 66]. Chan and Rosai expanded on the group of tumors designated as tumors of the neck showing thymic or related branchial pouch differentiation [67] that may be related to solid cell nest or thymus origin (see below intrathyroid thymic carcinoma). To date, a pure solid cell nest tumor has not been defined. However, exceptional thyroid neoplasms with cytomorphological and immunohistochemical features of main cells of solid cell nests have also been reported mimicking a basaloid neoplasm [68] (Fig. 5a). These basaloid cells disclosed expression of markers typical of the main cells of solid cell nests favoring a histogenesis link between this tumor and solid cell nests and also supporting a pathogenesis link between PTC and ultimobranchial body remnants [69] (Fig. 5b and c). In such a case, the possibility of a metastatic disease should be excluded after exhaustive search for a primary neoplasm elsewhere.

### Hyalinizing Trabecular Tumor

Hyalinizing trabecular tumor (HTT) is a solid, well-delineated thyroid neoplasm composed of large trabeculae of elongated or polygonal follicular cells admixed with prominent amounts of intertrabecular and intratrabecular hyaline (PAS-positive) material [2] (Fig. 5d). HTTs with invasive growth are termed as hyalinizing trabecular carcinomas. The tumor cells display nuclear alterations of PTCs including nuclear grooves and intranuclear pseudoinclusions. For this reason, such tumors have been linked to a variant of PTC by some experts. Mitotic activity, infiltration, and/or angioinvasion have been found only in rare malignant cases [70, 71].

By immunohistochemistry, the tumor cells are positive for thyroglobulin and TTF1. Positivity for galectin-3, HBME1, and CK19 can also be seen in some cases [2]. HTT is always negative for calcitonin and CGRP [2]. Ki67 shows characteristic membrane staining pattern (Fig. 5e), but the latter occurs only when the immunostaining is performed at room temperature [72]. The hyaline material is negative for amyloid and positive for type IV collagen (Fig. 5f) and laminin.

FNAB can also yield clues to the diagnosis of HTT. Hyaline material resembling amyloid can lead to an erroneous diagnosis of medullary thyroid carcinoma or amyloid goiter. Abundance of tumor cells with nuclear grooves and pseudo-inclusions makes it difficult to differentiate HTT from conventional PTCs. However, the absence of papillary structures and fibrovascular stalks associated with the hyaline material can raise the possibility of a HTT [73].

*RET/PTC* rearrangements have been detected in HTTs, but no *BRAF* nor *RAS* mutations have been observed [74]. Recent evidence suggests that *PAX8-GLIS3* and *PAX8-GLIS1* fusions appear to be a pathognomonic genetic alteration of these neoplasms [75].

## Tumors with Signet Ring Cell and Lipid Cell Change

### Signet Ring Cell Change in Follicular Tumors

Follicular tumors and hyperplastic thyroid nodules with signet ring cells are composed of tumor cells with large intracytoplasmic vacuoles that displace and compress tumor nuclei [2, 41] (Fig. 5g–i). Signet ring cells often contain large cytoplasmic vacuoles lined by microvilli or distended vesicles that are positive for mucin stains (positive for PAS and Alcian blue with pH 2.5). These tumors have been designated as signet ring cell mucinous adenoma or mucin-producing adenoma of the thyroid gland when there is neither invasion nor nuclear alterations of PTC. Thyroid carcinomas with synchronous abundant extracellular myxoid/mucoid matrix and signet ring cell change have been also reported [76].

Signet rings cells can be appreciated during cytological examination on FNAB smears [77, 78]. Nevertheless, the universal criteria used for the diagnosis of various forms of thyroid neoplasms are applicable to those with prominent signet ring cell change. Absence of TTF1 and monoclonal PAX8 and/or thyroglobulin suggests metastatic spread from stomach and breast.

Signet ring cells with eosinophilic inclusions have also been reported in secretory carcinoma of the thyroid gland. Intrathyroidal mammary analog secretory carcinoma (MASC) is composed of solid sheets and nests in a fibrovascular stroma, with cleft-like structures, cribriform areas, microcysts, and focal pseudopapillae and/or a few true papillae [78–81]. Tumor cells have abundant eosinophilic or vacuolated cytoplasm and monotonous round nuclei with clear nucleoplasm and conspicuous nucleoli. This infiltrative thyroid tumor shows expression for PAX8 and cytokeratin 19 and can mimic PTC, but the tumor cells are negative for thyroglobulin and show negativity (clone 8G7G3/1) or focal positivity (clone SPT24) for TFF1. MASC of the thyroid stains positively for mammaglobin, GCDFP-15, S-100 protein, and p63 and shows the *ETV6-NTRK3* translocation similar to its salivary gland or breast counterpart [78–81].

### Lipid-Rich Follicular Tumors

**Fig. 6:**
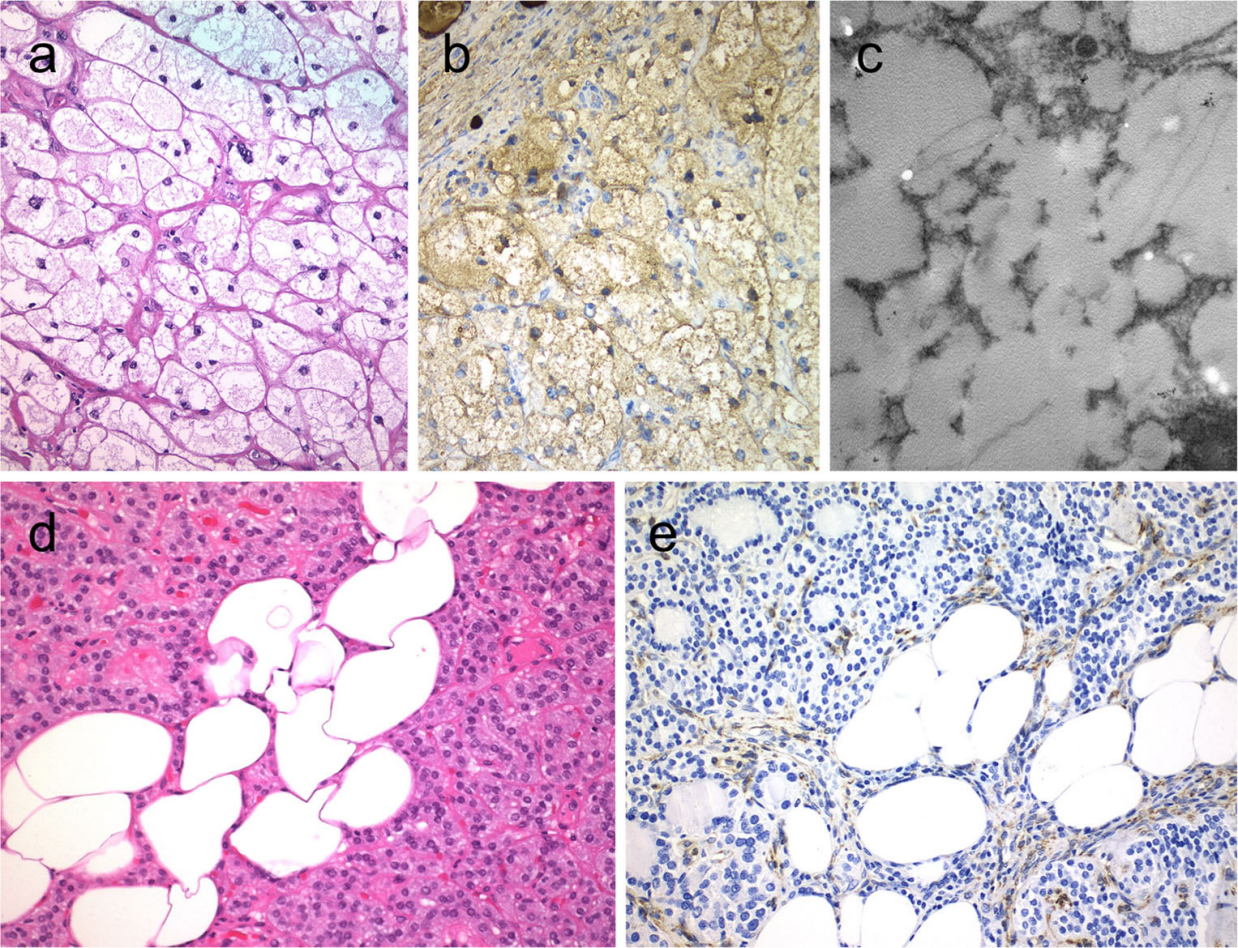
Lipid-rich follicular thyroid carcinoma (**a**) immunoreactive for thyroglobulin (**b**); the ultrastructural study evidenced numerous lipid vacuoles in the cytoplasm (ultrastructure) (**c**). Adenolipoma (lipoadenoma) in a patient with PTEN hamartoma tumor syndrome (**d**); there is negativity for PTEN protein in tumor cells while stromal cells (internal positive control) are positive (**e**)

Another subset of clear cell thyroid tumors is linked to intracytoplasmic accumulation of lipid droplets [76, 82]. Lipid-rich follicular cell-derived tumors often display microfollicular and/or solid pattern of growth. The majority of tumor cells have a peculiar clear, microvesicular, foamy cytoplasmic appearance (Fig. 6a–c). The size of the lipid vesicles is variable. The microvesicles can coalesce sometimes and lead to a signet ring-like appearance. The FNAB may be used to confirm lipid content using the oil red O stain. The lipid accumulation in follicular cells may indeed not be a result of a simple storage issue but a reflection of altered intracellular lipid metabolism [83]. The transition from protein synthesis to lipid synthesis is reflected in neoplastic cells progressing from non-clear to clear cell appearance [84].

Most lipid-rich follicular thyroid neoplasms are benign adenoma, but malignant counterparts have been defined in association with vascular and/or capsular invasion [1, 84]. Lipid-rich follicular tumors should not be mistaken for parathyroid lipoadenoma [85, 86] or thyroid adenolipoma (lipoadenoma), as well as rare FTCs and PTCs with stromal abundant adipose tissue deposition. Thyroid adenolipomas (Fig. 6d) are rare tumors in which adipocytes are intermixed between edematous thyroid follicles. These tumors have also been associated with the PTEN hamartoma tumor syndromes (Fig. 6e) [1]. Immunohistochemical biomarkers, especially thyroglobulin, TTF1, and PAX8, can help in confirming the thyroid follicular origin.

## Tumors with Mucin Deposition, Squamous Cell, or Squamous Cell-Like Features

### Mucinous Follicular Tumors

**Fig. 7:**
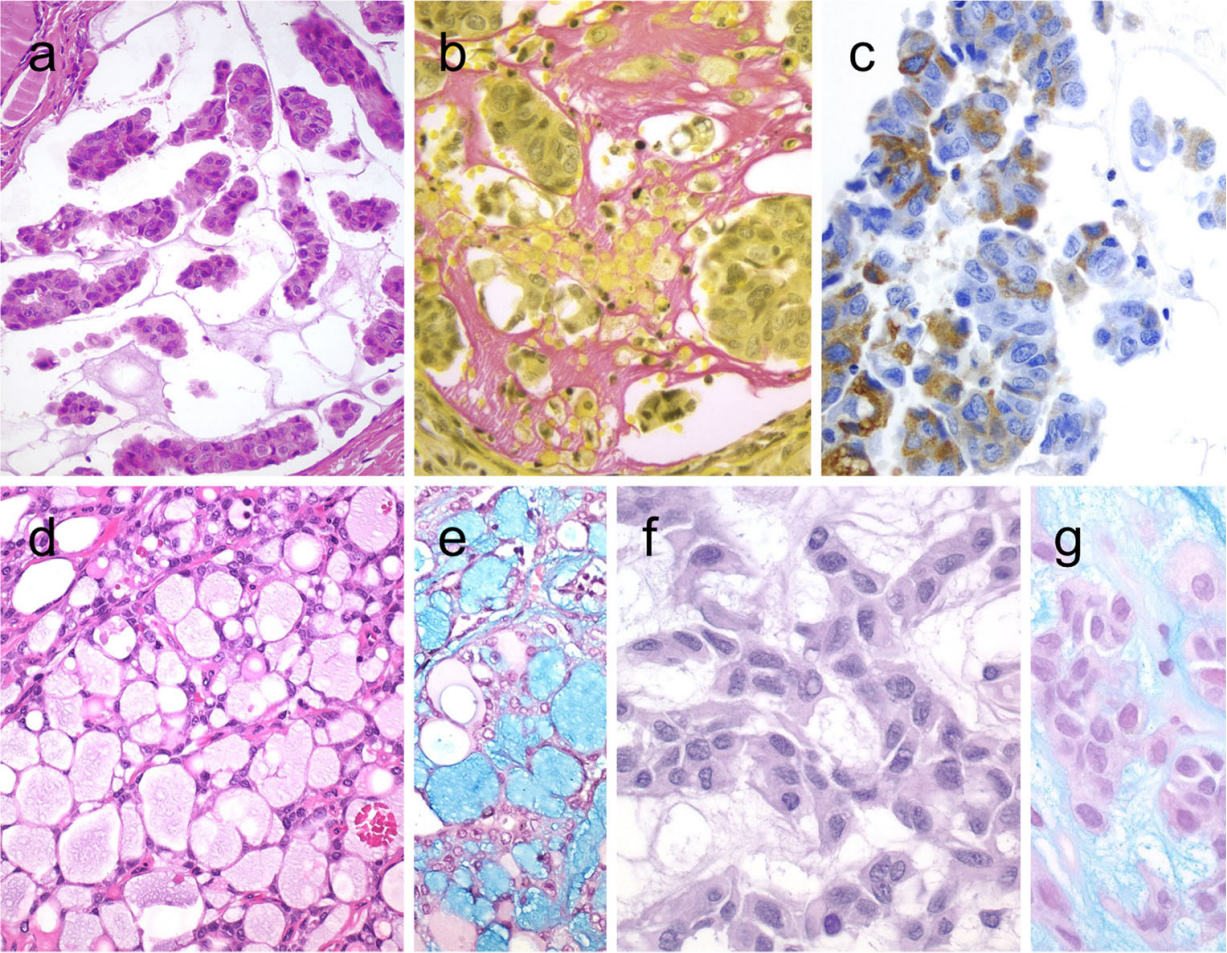
Mucinous thyroid carcinoma (**a**) showing abundant mucoid material mucicarmine positive (**b**); most tumor cells were positive for thyroglobulin (**c**). In this mucinous variant of follicular thyroid carcinoma (**d**), the follicles were distended and full of Alcian blue–positive mucinous material (**e**). Mucinous variant of papillary thyroid carcinoma (PTC) (**f**), the tumor showed ribbon, trabecular and/or follicular pattern, classic nuclear features of PTC and abundant mucoid stroma positively stained with Alcian blue (**g**)

Mucinous variants of FTC and PTC are composed of neoplastic follicular cells surrounded by extensive extracellular mucin deposition [2] (Fig. 7a–g). The overall features are somewhat identical to those of mucinous (colloid) carcinoma of various organs. However, the mucinous variants of FTC (Fig. 7d and e) are invasive tumors that are composed of follicular cells displaying an exclusive follicular architecture in the background abundant mucoid stroma, whereas mucinous variants of PTC often display nuclear alterations characterized by nuclear enlargement, oval and irregularly contoured nuclei with frequent intranuclear pseudoinclusions [1] (Fig. 7f and g).

The mucinous material stains positively with several histochemical stains including PAS (with and without diastase), the iron diamine method, mucicarmine, and Alcian blue (pH 2.5) [87–89] (Fig. 7b, e, and g). The presence of mucin antigens (MUC1, MUC2, MUC3, and others) alone does not specify this diagnosis. One should distinguish metastatic carcinoma in such tumors. The possibility of a metastatic tumor from lung, breast, digestive tract, or other organs should always be considered when dealing with a mucinous thyroid tumor (see below metastatic carcinomas). Nevertheless, positivity for thyroglobulin (Fig. 7c), TTF1, PAX8, and low-molecular-weight cytokeratins assists the distinction of mucinous thyroid carcinomas from metastatic mucinous carcinomas [89–91]. Thyroglobulin and TTF1 can be negative when dedifferentiation to anaplastic thyroid carcinoma occurs [91]. These tumors are almost always negative for calcitonin and CGRP. Positivity for p53 can be observed [90]. Mucinous thyroid tumors can also be encountered on FNAB specimens [91]. Therefore, the application of special stains can assist the diagnostician.

### Thyroid Tumors with Squamous Cell Change

**Fig. 8:**
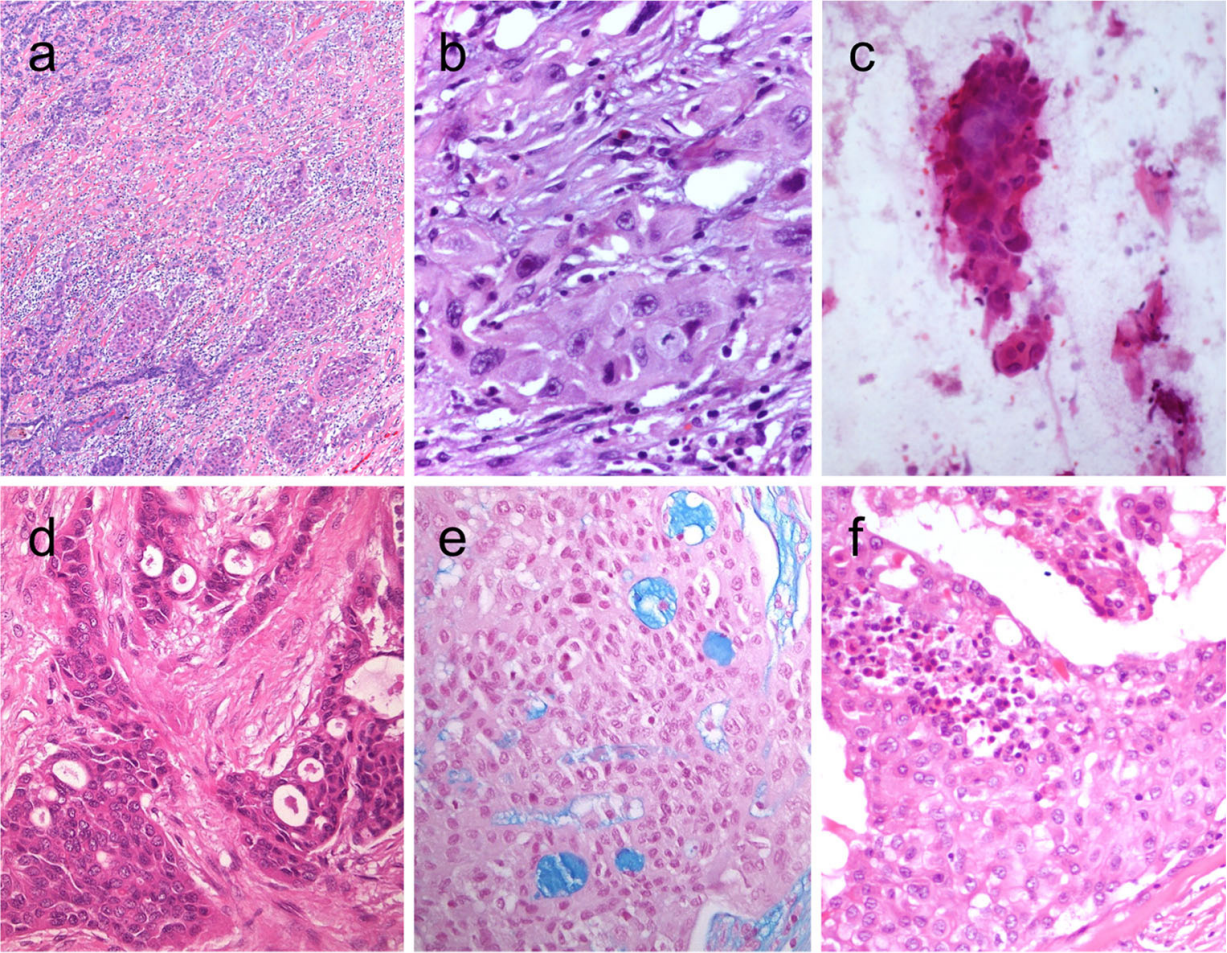
Squamous cell tumor examples that include extensive squamous metaplasia in PTC after fine needle aspiration biopsy (FNAB) (**a**), squamous cell carcinoma in the thyroid of putative secondary origin (**b**), and squamous cell carcinoma of the esophagus metastatic in the thyroid and diagnosed by FNAB (**c**). Mucoepidermoid carcinoma (**d**) composed by solid sheets of epithelial cells showing epidermoid cells and glandular spaces containing mucinous material positively stained with Alcian blue (**e**). Sclerosing mucoepidermoid carcinoma with eosinophilia showing epithelial cells richly infiltrated by eosinophils, lymphocytes, and plasma cells (**f**)

The occurrence of a squamous cell metaplasia is not unusual in all sorts of benign and malignant follicular cell-derived tumors [1, 2] (Fig. 8a–c). A squamous cell variant of medullary thyroid carcinoma has also been described [92]. Primary squamous cell carcinoma of the thyroid is extremely rare [2, 93]. In addition, anaplastic thyroid carcinomas can also display areas with squamous change and express PAX8 as also detected in primary squamous cell carcinoma of the thyroid. Because curative resection is more often possible in squamous cell thyroid carcinoma than in anaplastic thyroid carcinoma, the early detection of primary thyroid squamous cell carcinoma is important for achieving a curative surgical resection [93]. Furthermore, the presence of a squamous cell carcinoma in the thyroid requires exclusion of metastasis or direct invasion from adjacent head and neck squamous carcinomas. Diffuse positivity for TTF1 and PAX8 can be used to support the thyroid follicular origin. P53 overexpression and *BRAF* mutation can also be detected in primary squamous cell thyroid carcinoma [93]. FNAB is sensitive to detect the malignant nature of the nodule in only 79% of cases of primary squamous cell carcinoma of the thyroid [93].

### Mucoepidermoid Carcinoma

Mucoepidermoid carcinoma is a rare malignant epithelial thyroid tumor showing a combination of mucin-producing cells and epidermoid cells [2, 94] (Fig. 8d and e). In about half of these neoplasms, an association with PTC (conventional, follicular, columnar cell, or tall cell variants) has been reported [2, 95, 96]. In some cases (with or without PTC), poorly differentiated and/or anaplastic carcinoma component may be seen, and such cases have been linked to a worse prognosis [97]. FNAB specimens occasionally result in accurate diagnosis of these tumors [98, 99].

By immunohistochemistry, the tumor cells are positive for high- and low-molecular-weight keratins and usually positive for thyroglobulin, TTF1, and PAX8. Epidermoid cells are positive for p40 and p63, whereas mucinous cells and duct-like structures are positive for CEA (polyclonal) [94, 100, 101]. There is also mRNA expression of the *TTF1*, *TTF2*, *PAX8*, *SLC5A5*, and *TPO* genes [102].

Recent evidence suggests the occurrence of *CRTC1-MAML2* fusion in 1 of 3 tested mucoepidermoid carcinoma [103]. *RET/PTC* rearrangements were found in combined PTC and mucoepidermoid carcinoma, whereas no *BRAF* or *RAS* mutations were detected in mucoepidermoid carcinomas [96]. More recently, a targeted next-generation sequence analysis of a case of concurrent mucoepidermoid and PTC revealed a monoallelic germline *MUTYH* mutation combined with somatic mutations of the *BCOR* and *MSH2* genes in the mucoepidermoid carcinoma and PTC components, respectively [104]. Although some solid cell nests can contain mucocytes and cystic lumina, the main cells of solid cell nests are negative for thyroglobulin [63, 65, 105].

### Sclerosing Mucoepidermoid Carcinoma with Eosinophilia

Sclerosing mucoepidermoid carcinoma with eosinophilia (SMECE) is a rare malignant epithelial thyroid neoplasm [2]. SMECE is characterized by small nests and/or strands of tumor cells with both squamous and mucinous differentiation embedded in a sclerotic or fibrohyaline stroma with variable lymphocytes, plasma cells, and generally fewer eosinophils [106] (Fig. 8f). In some cases, glycogen-rich clear epidermoid cells can predominate.

By immunohistochemistry, SMECE is positive for keratins (including CK19), p63, CD10, and galectin-3; around 50% of cases are positive for TTF1 and occasionally for PAX8 (polyclonal) [107, 108]. They are usually negative for thyroglobulin, but rare focal positivity (query diffusion artifact) for thyroglobulin has also been reported [107–109]. SMECEs are always negative for calcitonin. p53 is occasionally seen in epidermoid cells [107].

*BRAF*^V600E^ mutation was detected in two cases [110], and an *APC* gene variant of uncertain significance was also found in another case of SMECE [111]. Compared with conventional mucoepidermoid carcinomas, SMECEs are associated with more aggressive tumor biology. These tumors also show increased association with dense sclerosis, Hashimoto thyroiditis, and prominent eosinophilia. Coincidental cases of SMECE and PTC are rare [107].

### Metastatic Carcinomas in the Thyroid Gland

Thyroid metastases have been reported in 1.4–3% of all patients who have surgery for suspected thyroid cancer [112]. They account for about 2% of all thyroid malignancies and represent 2.3–7.5% of patients submitted to FNAB [113]. In autopsy studies, the lung have been reported to be the most common source of metastatic disease in the thyroid gland, whereas in clinical series, kidney accounts for the most common origin [112]. Metastatic primaries followed by kidney include the lung, breast, gastrointestinal tract, head and neck, skin, lymphoid neoplasms, and gynecological tract. A higher frequency of sarcoma metastasizing to the thyroid has been recently reported [114, 115].

**Fig. 9:**
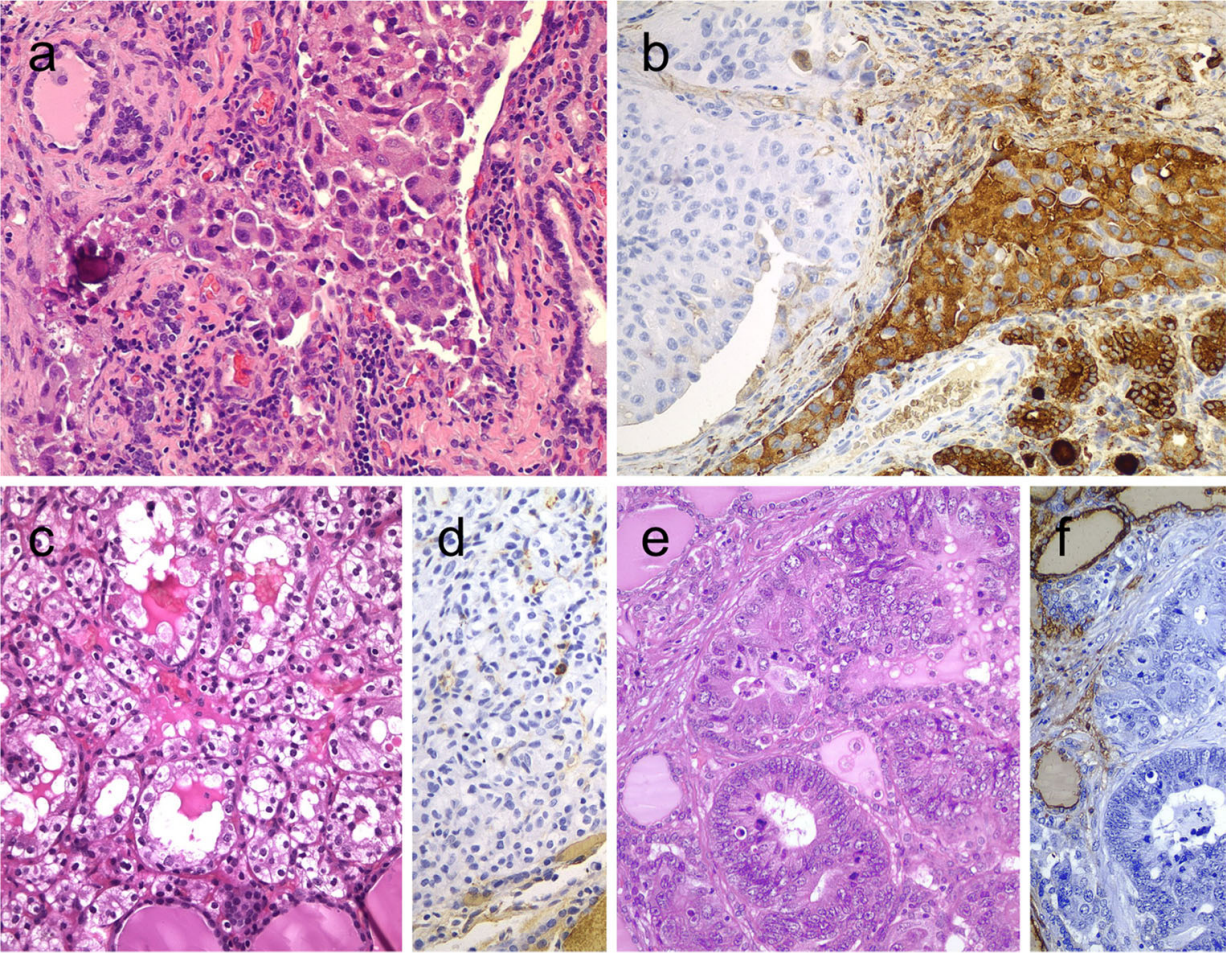
Metastatic carcinomas in the thyroid gland. Thyroid metastasis from lung adenocarcinoma (**a, b**). Some metastatic tumor cells (right) are positive for thyroglobulin due to diffusion artifact and should not be overinterpreted as positive (**b**). Metastatic clear cell renal carcinoma (**c**), metastatic renal cells are negative for thyroglobulin (**d)**. Colonic adenocarcinoma metastatic to the thyroid gland (**e**); the thyroid tissue is positive for thyroglobulin while the metastatic adenocarcinoma is negative (**f**)

Metastatic carcinomas can display various cytomorphology or mucin deposition that can simulate various neoplasms [116]. Immunohistochemistry plays an important role for an accurate diagnosis. One should be careful not to interpret thyroglobulin diffusion staining as a sign of follicular thyroid origin (Fig. 9a and b). Renal clear cell carcinoma and some breast carcinomas can pose diagnostic challenge [2, 114], and some tumors can give rise to metastases within an existing thyroid carcinoma [44] (Fig. 9c and d). Metastasis to thyroid are more frequent in glands that have underlying nodular or inflammatory disease [115], and they can also appear within primary benign or malignant thyroid neoplasms (tumor-to-tumor metastasis), including NIFTPs [117–119].

Metastatic adenocarcinoma of the lung (TTF1+, napsin A+, PAX8-), colorectal carcinoma (β-catenin+, CDX2+, CK20+, SATB2+, PAX8-, and often TTF1-) or endometrial carcinoma (PAX8+, often TTF1-) can also simulate primary thyroid carcinomas (with a papillary and/or follicular pattern), but metastatic carcinomas are all negative for thyroglobulin [2, 112, 117] (Fig. 9a-f). The expression of napsin A is common in thyroid tumors [120].

In particular, metastatic squamous carcinomas should not be mistaken for a direct extension of a primary carcinoma from the larynx or esophagus. From this perspective, as defined by Dr. LiVolsi and her coworkers, the distinction of anaplastic spindle cell squamous carcinoma of the thyroid is of interest [121]. The latter is often seen in association with tall cell variant PTCs and can sometimes simulate a primary upper aerodigestive tract squamous cell carcinoma involving the thyroid gland. Therefore, the use of appropriate biomarkers can help in the confirmation of thyroid follicular origin.

Molecular testing could be useful in the workup of selected cases [122]. Lobectomy or total thyroidectomy is particularly considered in isolated tumors and slow-growing metastasis to thyroid such as those primaries from the kidney or breast [43, 113]. A recent meta-analysis showed that surgery increased both disease-free and overall survival in patients, even if accompanied by disseminated tumor, when compared with chemotherapy or local radiotherapy [113].

## Tumors with Predominant Spindle Cell Growth Pattern

### Spindle Cell Follicular Tumors

Follicular nodular disease, follicular adenoma, PTC, FTC, and medullary thyroid carcinoma can disclose focal or diffuse spindle cell phenotype [123]. The differential diagnosis between these tumors must follow the criteria of the respective non-spindle cell variants.

**Fig. 10:**
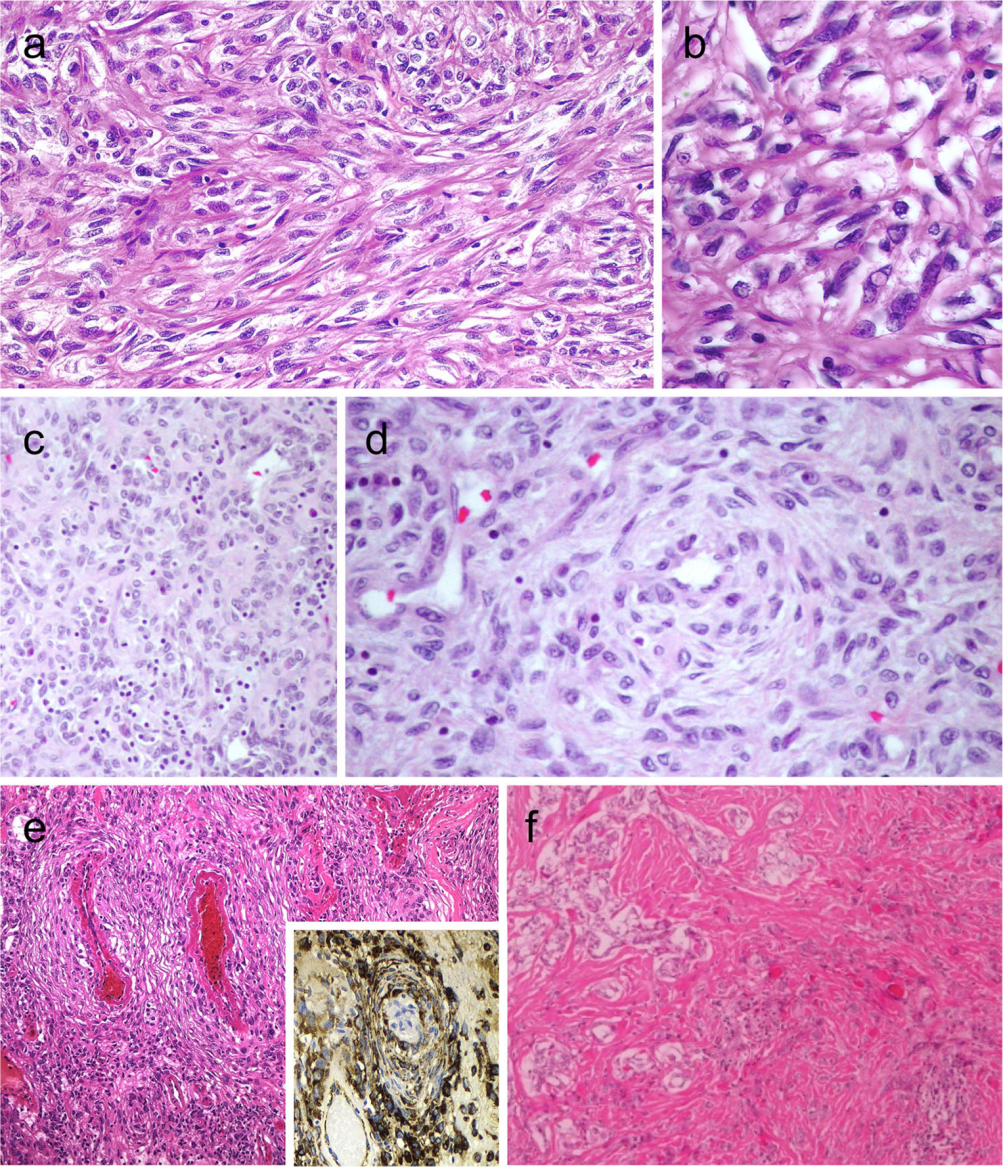
Spindle cell variant of papillary thyroid carcinoma (PTC) showing spindle cells with typical PTC nuclei (**a** and **b**). Meningioma-like follicular adenoma (**c** and **d**), the typical arrangement of spindle to ovoid cells in a whorled pattern may give the impression one is dealing with a vascular tumor. Pericytic-like follicular adenoma (**e**) is characterized by a proliferation of spindle follicular cells concentrically arranged around vessels; the follicular nature of the tumor cells could be confirmed by the positivity for thyroglobulin (inset), thyroperoxidase, TTF1 and cytokeratins but negativity for calcitonin and CD31. PTC with fibromatosis/fasciitis-like stroma with both stromal and PTC component (**f**)

Spindle cell variants of PTC are composed of tumor cells that are arranged in bundles [124] (Fig. 10a). Frequent nuclear grooves and less frequently pseudoinclusions also feature such tumors (Fig. 10b). Follicular structures may be seen at the periphery of the tumor.

The cellular origin of spindle cell tumors in the thyroid gland should be confirmed using immunohistochemistry. Combined expression for PAX8 and TTF1 distinguishes follicular epithelial origin. Thyroglobulin expression may be only focally observed in some cases.

Spindle cell follicular thyroid tumors should also be distinguished from benign conditions such as thyroiditis and post-FNAB-related reactive changes, as well as from various neoplasms including solitary fibrous tumor, spindle cell hemangioma, spindle cell variant of medullary thyroid carcinoma, and intrathyroid thymic–related neoplasms with spindle cell change (e.g., SETTLE and intrathyroidal thymic carcinoma) [125].

### Meningioma-Like and Pericytic-Like Follicular Adenoma

The meningioma-like tumor of the thyroid or meningioma-like follicular adenoma is a spindle cell tumor that is considered a cytomorphological variant of follicular adenomas [126]. The typical arrangement of bland-looking spindle to ovoid cells in a whorled pattern around blood vessels may also give the impression that one is dealing with vascular tumors of the pericytic type (Fig. 10c and d). Positivity for TTF1, PAX8, and thyroglobulin and coexistence of spindle cells with well-differentiated follicles support this diagnosis.

Pericytic-like follicular adenoma has also expanded cytomorphological spectrum of follicular thyroid neoplasms [1] (Fig. 10e). The follicular epithelial origin of this tumor should be confirmed using immunohistochemistry including positivity for thyroglobulin (Fig. 10e, inset), thyroperoxidase, TTF1, PAX8, and cytokeratins (clone AE1/AE3). These tumors are negative for negative for calcitonin, CD31, and CD34.

## Tumors with Peculiar Biphasic Pattern

### Papillary Thyroid Carcinoma with Desmoid-Type Fibromatosis

PTCs with fibromatosis/fasciitis-like stroma are now designated as PTCs with desmoid-type fibromatosis [127]. These are biphasic tumors that are composed of abundant cellular PTC component admixed with spindle cells with benign stroma resembling nodular fasciitis or desmoid-type fibromatosis [2, 128] (Fig. 10f).

The stromal component expresses nuclear beta-catenin and cytoplasmatic smooth muscle actin and lacks cytokeratins and TTF1. Nuclear detection of SOX11 can be an alternative diagnostic tool for evaluating tumors where nuclear expression for β-catenin is ambiguous [129].

PTCs with fibromatosis/fasciitis-like stroma should be distinguished from anaplastic thyroid carcinomas. Lack of nuclear beta-catenin expression in the stroma, lack of follicular epithelial differentiation, positivity for PAX8 and/or TTF1, and presence of increased proliferative activity are features that can help in the distinction of anaplastic thyroid carcinomas.

### Carcinoma of the Thyroid with Ewing Family Tumor Elements (CEFTE)

**Fig. 11:**
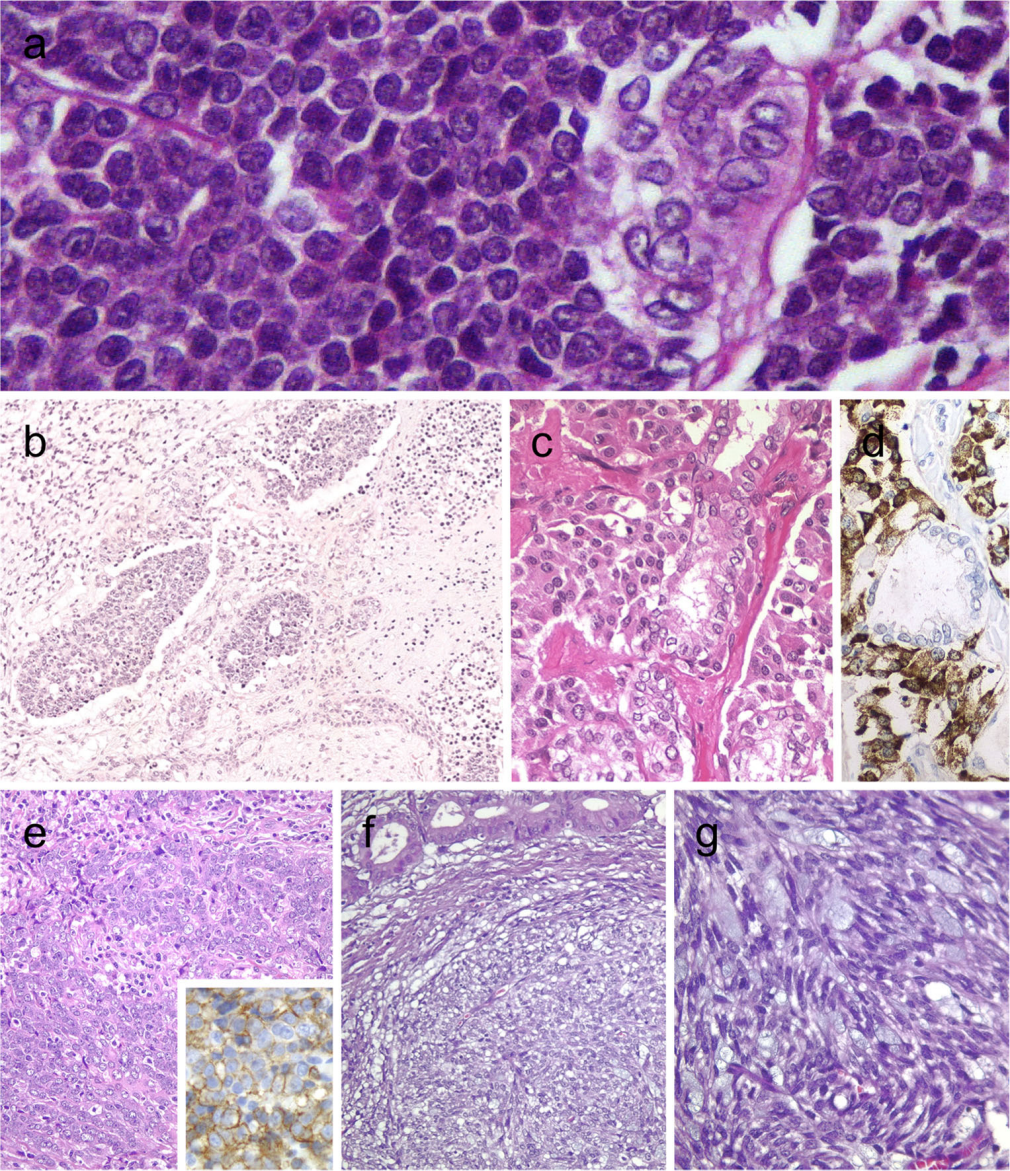
Carcinoma of the thyroid with Ewing family tumor elements (CEFTE) disclosing solid nests of small cells with regular, round nuclei, and nests of papillary thyroid carcinoma (PTC) (**a**). This case is from a 17-year-old female patient with bilateral involvement of the thyroid by a malignant thyroid teratoma (**b**); the tumor discloses nests of small cells, rich stroma with chondroid appearance and an epithelial-tubular component. Mixed medullary and papillary thyroid carcinoma (**c**); the medullary thyroid carcinoma component stained positively for calcitonin mRNA while the PTC (follicular variant) component was negative (**d**). Intrathyroid thymic carcinoma (ITC) also known by the acronym (CASTLE) showing positivity for CD5 (inset) (**e)**. Spindle epithelial tumor with thymus-like differentiation (SETTLE) is a lobulated tumor composed of spindle cells and epithelioid cell component with glands, mucinous cysts, and/or squamous nests (**f** and **g**)

The carcinoma of the thyroid with Ewing family tumor element (CEFTE), also designated as adamantinomatous-like Ewing tumor, is an invasive primary of the thyroid that resembles Ewing sarcoma of the soft tissue (Fig. 11a). CEFTEs are usually large tumors that occur in young patients. These tumors have been linked to a favorable prognosis [130, 131].

CEFTEs are composed of small cells that show strong and diffuse immunoreactivity for p63, CD99, and cytokeratins. TTF-1, thyroglobulin, and calcitonin are typically negative.

At a molecular level, *EWSR1/FLI1* rearrangement is pathognomonic as the latter has been detected in all reported tumors. The association of *EWSR1* rearrangements in PTC also supports the hypothesis that CEFTEs probably represent a “trans-dedifferentiation” phenomenon in PTCs [132].

CEFTEs need to be distinguished from small cell variants of medullary thyroid carcinoma and poorly differentiated thyroid carcinoma. Absence of p63 and CD99 makes unlikely the possibility of CEFTEs. However, the distinction of CEFTE from a typical Ewing tumor is a real challenge. Negativity for vimentin and presence of epithelial differentiation and the co-existence of PTC component support the diagnosis of CEFTE. The favorable prognosis of CEFTE is also a distinct feature. Data on preoperative cytological features of CEFTE are scant. In one reported case, the FNAB resulted in a Bethesda V cytology consistent with undifferentiated thyroid carcinoma [133].

Furthermore, teratomas with somatic malignancy also pose diagnostic challenges. Unlike CEFTEs, malignant thyroid teratomas are biologically very aggressive small cell neoplasms that express SALL4 and Glypican3 and are negative for p63 [134, 135]. Unlike CEFTEs, this aggressive primitive multiphenotypic malignancy showing organotypical elements and frequent *DICER1* alterations has been recently referred to as the term “thyroblastoma” [135] (Fig. 11b).

### Mixed Medullary and Follicular Thyroid Carcinoma

Mixed medullary and follicular thyroid carcinomas (MMFTC) are rare primary malignant thyroid neoplasms that are composed of two distinct tumor components with distinct morphological and immunohistochemical evidence of C cell and follicular cell lineages within the same lesion [2]. The proportion of each of the two lineages can vary, and there is no well-established cutoff point [136]. The existence of synchronous medullary thyroid carcinoma and follicular cell-derived carcinomas in close proximity but without intermixing is considered “collision tumors,” and should not be classified as MMFTC [137].

The histology of the medullary thyroid carcinoma component of MMFTC is not different than that of the conventional medullary thyroid carcinoma (Fig. 11c). The follicular cell component is usually represented by the follicular variant of PTC (Fig. 11c). However, rare examples of conventional PTC, follicular thyroid carcinoma, oncocytic or poorly differentiated carcinoma, and undifferentiated thyroid carcinoma have been documented [138]. Metastases can originate from both tumor components (this also supports the diagnosis of a mixed tumor); however, this can also consists of one component [139].

The diagnosis of MMFTC requires the use of immunohistochemistry to confirm the dual nature of C cell–derived (positive for calcitonin, CGRP, and monoclonal CEA and negative for monoclonal PAX8) and follicular (positive for thyroglobulin and monoclonal PAX8) [39] (Fig. 11d). TTF1 is positive in both components. PAX8 expression in medullary thyroid carcinoma depends on the antibody used [39]. Although calcitonin and thyroglobulin are generally not simultaneously expressed by the same cell, dual expression of both markers has been detected in rare cases [138]. This dual differentiation has been evidenced by mRNA and ultrastructurally [140]. FNAB findings are usually consistent with a diagnosis of medullary thyroid carcinoma [141].

In some MMFTCs, the follicular component was found to be oligo-/polyclonal and therefore possibly hyperplastic rather than neoplastic [142]. The follicular cells may have grown into the medullary thyroid carcinoma, after acquiring some molecular alterations, being “hostage” of the true neoplastic medullary thyroid carcinoma component [138, 143]. Somatic *RET* mutations have been detected exclusively in the medullary thyroid carcinoma component of MMFTC. Only a few cases have occurred in the setting of multiple endocrine neoplasia type 2 [142].

### Intrathyroid Thymic Carcinoma

Intrathyroidal thymic carcinomas (ITC) are rare malignant thyroid tumors with thymic epithelial differentiation [2, 67, 144]. Also known by the acronym CASTLE (carcinoma showing thymus-like elements) [145], ITC is the malignant counterpart of ectopic thymoma of the thyroid [146, 147] (Fig. 11e). It has been postulated that the ITC arises either from ectopic thymus or remnants of branchial pouches which retain the potential to differentiate along the thymic line [67].

The three ITC histological subtypes include keratinizing squamous cell carcinoma type, non-keratinizing basaloid cell carcinoma (lymphoepithelioma-like) type, and neuroendocrine carcinoma type are equivalent to those of the mediastinal thymic carcinoma [147]. Most ITCs are made up of squamous cell carcinoma lobes separated by a fibrous stroma densely infiltrated by lymphocytes and plasma cells (lymphoepithelioma-like carcinoma of the thyroid). Tumor cells are polygonal with distinct nucleoli and ill-defined cell borders. Occasional single-cell keratinization or stratification of keratinizing tumor cells can be seen, but nuclear atypia is mild and mitoses uncommon. Hassall corpuscles may be seen at the periphery of the tumor.

By immunohistochemistry, neoplastic cells are positive for CD5 (Fig. 11e, inset), p63, KIT (c-KIT, CD117), polyclonal PAX8, high-molecular-weight cytokeratins, a wide-spectrum of cytokeratins (clone AE1/AE3), CEA, GLUT-1, EGFR, calretinin, p53, bcl-2, and mcl-1 and are negative for thyroglobulin, TTF1 (except clone SPT24 can show scattered positivity), calcitonin, and CD45RB (LCA) [147–149]. There are also scattered S-100A9-positive cells, similar to that those of thymoma.

No association with EBV has been found [147]. Unlike eutopic thymic carcinomas, *TERT* promoter mutations have been identified in a subset of ITCs [150].

The cytological features of ITC closely resemble those of metastatic nasopharyngeal carcinoma. When compared with nasopharyngeal carcinomas, ITCs are relatively low-grade tumors with a Ki67 index of approximately 10–30% and show diffuse positivity for CD5, whereas primary squamous carcinomas tend to display more keratinization, a higher histological grade with a Ki-67 labeling index exceeding 50%, and negativity for CD5 and S-100A9. Anaplastic thyroid carcinomas (including those cases with squamous differentiation) show pronounced pleomorphism, atypical mitotic figures, tumor necrosis, and negativity for CD5. Neuroendocrine differentiation (positivity for chromogranin and synaptophysin) has been detected in some ITCs with simultaneous positivity for CD5 and negativity for calcitonin [147, 149].

### Spindle Epithelial Tumor with Thymus-like Differentiation

Spindle epithelial tumor with thymus-like differentiation (SETTLE) is a malignant primary of the thyroid that occurs in young patients and children and is thought to be derived from the branchial pouches or intrathyroid thymic remnants [2, 67].

SETTLE is a lobulated tumor, usually limited by a capsule with fibrous septa. These tumors are composed of bland spindle cells and epithelial (or epithelioid) cells that may form tubules with mucinous cysts, small papillae, trabeculae, or squamous nests (Fig. 11f and g).

By immunohistochemistry, the spindle cell component expresses low- and high-molecular-weight cytokeratins, p63, vimentin, and CD99. Thyroglobulin, calcitonin, and TTF1 are usually negative in both components.

The differential diagnosis of SETTLE includes spindle cell tumors described above. The distinction between SETTLE and synovial sarcoma can be made only after the exclusion of the t(X,18) rearrangement which is characteristic of synovial sarcoma.

## Conclusion

Thyroid pathology encompasses a wide spectrum of cytomorphological entities that can pose diagnostic challenges. Knowledge on the wide spectrum of clinicopathological characteristics and appropriate use of ancillary biomarkers often assist diagnosticians when rendering an accurate diagnosis.
